# Long Head of the Biceps Pathology Combined with Rotator Cuff Tears

**DOI:** 10.1155/2012/405472

**Published:** 2012-11-19

**Authors:** Konstantinos Ditsios, Filon Agathangelidis, Achilleas Boutsiadis, Dimitrios Karataglis, Pericles Papadopoulos

**Affiliations:** 1st Department of Orthopaedics, G. Papanikolaou General Hospital, Aristotle University of Thessaloniki, Exohi, 57010 Thessaloniki, Greece

## Abstract

The long head of the biceps tendon (LHBT) is an anatomic structure commonly involved in painful shoulder conditions as a result of trauma, degeneration, or overuse. Recent studies have pointed out the close correlation between LHBT lesions and rotator cuff (RCT) tears. Clinicians need to take into account the importance of the LHBT in the presence of other shoulder pathologies. This paper provides an up-to-date overview of recent publications on anatomy, pathophysiology, diagnosis, classification, and current treatment strategies.

## 1. Introduction

Evolution in shoulder arthroscopy has brought into light painful conditions of the long head of the biceps tendon (LHBT) that need treatment [1]. In order to treat these conditions a clinician has to be able to identify them. In recent years there are numerous publications on the anatomy, histology, imaging, classification, clinical evaluation, and treatment of LHBT pathology ranging from tendinitis and instability to tears. There is also a growing tendency to consider rotator cuff and LHBT pathology closely related. Since these conditions either from trauma, degeneration, or overuse can occur simultaneously, they need to be identified and treated at the same time. This paper is a review of the literature with a focus on the relation between LHBT pathology and RCT.

## 2. Anatomy

The LHBT arises within the shoulder joint capsule. It is attached to the supraglenoid tubercle of the scapula and has two portions: one intra-articular and one extra-articular. The intra-articular part is more wide and flat compared to the extra-articular one which is rounder and smaller. The total length of the tendon is approximately 9 cm while its diameter is 5 to 6 mm [2]. The LHBT exits the glenohumeral joint through the rotator interval—a triangular portion of the shoulder capsule between the supraspinatus and subscapularis tendons—and enters into the bicipital groove where it is held in place by the transverse humeral ligament and the pectoralis major which crosses in front of the groove. However the mere existence of this ligament has been questioned [3].

The superior glenohumeral ligament (SGHL) and the coracohumeral ligament (CHL) seem to play an even more important role in stabilizing the tendon. The SGHL attaches to the superior most tendon of the subscapularis forming a fold of loose connective tissue. The CHL is considered to provide tension to the SGHL [4, 5]. These two ligaments along with the subscapularis tendon form the biceps pulley or sling [6] which stabilizes the LHBT from anteromedial dislocation. Part of the CHL insertion passes fibers behind the LHBT at the greater tuberosity and blend with fibers of the supraspinatus tendon to form a posterior pulley of the LHBT [7]. 

The blood supply of the tendon comes mainly from branches of the brachial artery from the musculotendinous side and osteotendinous derived vessels from the insertion side. There is a consistent hypovascular area 1, 2 to 3 cm from the tendon origin, possibly explaining the susceptibility of this area to rupture. The classic knowledge that the blood supply comes from the circumflex humeral artery is not consistently seen and is a less common variation [8]. The anterior superficial part of the tendon is better vascularized whereas the lateral, posterior, and medial side especially the part of the tendon adjacent to bone appears to be avascular.

Alpantaki et al. showed that the LHBT is innervated by a network of sensory sympathetic fibers, which may play a role in the pathogenesis of shoulder pain [9]. However, the innervation of the tendon is not well documented.

Several anatomic variations of the intra-articular part of the LHBT exist, and some types seem to be associated to labral pathologies [10, 11]. Anatomic variations have been described concerning the origin of the tendon with more than 50% being from the posterior labrum but with unknown clinical relevance [12].

## 3. Pathophysiology Classification of Long Head Biceps Tendon Lesions Concomitant with Rotator Cuff Tears

In 1990, Patte et al. observed the analgesic effect of spontaneous rupture of the LHBT and proposed simple arthroscopic tenotomy as a palliative treatment in patients with irreparable rotator cuff tears (RCTs) [13]. Full-thickness rotator cuff tears are commonly associated with lesions of the long head of biceps, contributing to anterior shoulder pain and forward flexion dysfunction [14, 15].

In the early 80s Neviaser correlated the severity of rotator cuff disease with the extent of inflammatory changes of the LHBT [16], while Miller and Savoie found that 74% of individuals with full-thickness rotator cuff tears had associated intra-articular lesions, with the labral tears being the most common disorder [17].

In an effort to understand the pathophysiology of these various lesions, Refior and Sowa [18] proposed a model of repetitive traction, friction, and glenohumeral rotation possibly due to upward migration of the humeral head in patients with an already dysfunctional rotator cuff. Pressure and shear forces can occur on the tendon at distinct, anatomically narrow sites resulting in degenerative changes as fibrosis, thickening, collagen disorganization, scar tissue, and adhesions development [19].

Additionally, Ahmad et al. [20] observed that diseased LHB tendons, in cadavers, have an increased cross-sectional area and a higher average load to failure compared with the healthy tendons. Moreover, they reported a relative stenosis of the bicipital groove in these shoulders enhancing the hypothesis of the parallel degeneration model of the LHBT with the rotator cuff tendons.

According to the literature, no classification system has yet been established that describes LHBT lesions as a type of biceps pathology associated with RCTs. The lesions may vary in degree from tendinitis and delamination to subluxation or dislocation over the medial rim of the bicipital groove and even to joint entrapment because of hypertrophy or hourglass deformity [21–24]. Chen et al. [25] proposed a simplified classification system of LHBT lesions combined with RCTs. The study included five types of lesions: tendinitis, subluxation, dislocation, partial tear, and complete ruptures. More recently the same main author [26] after reviewing 176 shoulders with complete, full-thickness RCTs defined six types of LHBT pathology: tendinitis (Type I), subluxation (Type II), dislocations (Type III), partial tears (Type IV), complete tears (Type V), and SLAP lesions (Type VI). Nevertheless, these retrospective publications need longer followup and future studies to confirm previous observations and to enhance proper surgical management.

## 4. Clinical Presentation and Examination

The clinical presentation of a patient with an LHBT pathology is similar to that of a patient with rotator cuff pathology that being tendinitis or tear. The patient reports anterior shoulder pain and impaired function as a result of either overuse or acute trauma. In the special case of overhead throwing athletes a snap during the followup through phase can indicate a lesion of the tendon, either an tear or a SLAP lesion. The Popeye sign, a lump on the lateral side of the arm from retraction of the tendon and ecchymosis, can be present.

SLAP lesion is a relatively new condition added to the shoulder pathology as a result of evolution in shoulder arthroscopy [1]. Since then various tests have been developed in an effort to associate clinical findings with underlying LHBT pathology including tendinitis, tear of the tendon, and SLAP lesions. The Yergason's test is positive when pain is present with resisted supination while the elbow is fixed in 90 degrees of flexion. For Speed's test the patient's elbow is extended, forearm supinated and the humerus in 90 degrees of forward flexion. The examiner resists humeral forward flexion and the test is positive when pain is present in the bicipital groove. Other tests such as the Neer impingement test, the Hawkins sign, Jobe's test, and O' Brien's test are overlapping and can be positive in various shoulder conditions like rotator cuff tears and tendinitis, impingement, and acromioclavicular arthrosis. Unfortunately none of the available tests can be considered useful in the clinical setting. Holtby and Razmjou [27] have shown that Yergason's and Speed's tests although they had specificity of 79% and 75%, they had sensitivity of 43% and 32% which is unacceptably low. Two reviews [28, 29] following chronologically this publication agree that although there are a few studies on various tests they cannot be considered valid because of serious shortcomings in their methodology. 

Injection of the bicipital groove with local anesthetic and corticosteroid solution can help differentiate LHBT tendinitis from other causes of anterior shoulder pain. A subacromial injection will relieve pain caused by impingement, and an intra-articular injection will be diagnostic and therapeutic if an SLAP tear is present [30]. Accuracy of such injections especially in the bicipital groove is improved under ultrasound guidance [31]. 

## 5. Imaging

Imaging for LHBT pathology should start with routine plain radiographs of the shoulder (anteroposterior, lateral, and axial views), mainly to rule out glenohumeral degeneration and acromioclavicular arthrosis and bony abnormalities that can cause impingement.

Ultrasound is inexpensive but is highly an operator-dependent technique. It can have high diagnostic value for the detection of tear, subluxation, or dislocation of the LHBT but it is not sensitive for the detection of partial thickness tears or tendinitis [32, 33]. 

MRI allows excellent visualization of the superior labral complex, the biceps tendon, the bicipital groove, and the presence of any bony osteophytes. As a result it can diagnose partial and complete tears of the LHBT tendon, SLAP lesions, dislocations of the tendon, and any associated pathology of the rotator cuff. However, the quality of MRI studies is not consistent. As a result MRI's findings correlate poorly with arthroscopic findings particularly in the diagnosis of partial thickness tears and tendinitis [34]. MRI arthrography on the other hand seems to be able to diagnose more accurately these conditions and especially biceps pulley lesions, tendinitis, and SLAP lesions [6, 35, 36]. Recent papers tend to raise the awareness that LHBT pathology is highly associated with rotator interval and biceps pulley lesions and that the presence of any finding in one of those structures mandates careful examination for hidden lesions in other structures [6].

## 6. Surgical Management

Surgical intervention of LHBT pathology is dependent on clinical presentation, symptoms' duration, the failure on conservative treatment, and mainly the coexistence of rotator cuff pathology. Surgical management indications are also partial-thickness tear of the LHBT or even LHBT subluxation in the setting of a subscapularis or biceps pulley tear [37, 38]. Despite the exact pathology, the LHBT is known to be a persistent pain generator in most cases of rotator cuff disease or impingement processes [39, 40], and surgical intervention is often indicated.

However, debate continues regarding the most suitable surgical treatment, with both tenotomy and tenodesis leading to favorable results in various reports [41, 42]. Different studies have reported widely variable differences in the clinical outcomes of these two surgical methods of treatment, and no clearcut advantage of any one of those two treatment modalities has been proven to date [43–46]. 

### 6.1. Biceps Tenotomy

Biceps tenotomy is a relatively simple and reproducible technique. Tenotomy involves division of the long head of the biceps tendon at its proximal insertion at the supraglenoid tubercle; this allows the tendon to retract away from the joint into the bicipital groove. In cases of hourglass deformity the biceps does not retract as a result of the enlargement, and approximately 1-2 cm of the tendon has to be removed. Usually this technique provides predictable pain relief and does not alter the postoperative rehabilitation program after a combined rotator cuff repair [41, 47].

However, it may result in a cosmetic defect (Popeye Sign, 3% to 70%), fatigue, or cramping pain or even in loss of elbow supination strength [15, 47, 48], possibly leading to a worse functional outcome. Popeye sign is less likely to cause discomfort problems to older persons or to those with obese arms. Additionally, fatigue cramping (38%) is more frequent in younger patients, aged <40 years old [47].

### 6.2. Biceps Tenodesis

Biceps tenodesis is performed in order to maintain the length-tension relationship of the biceps muscle and consequently prevent the muscle atrophy and preserve the normal contour. For this reason many authors believe that tenodesis should be used in younger, active patients, athletes, and laborers with combined LHBT and rotator cuff pathology. However, controversy remains about the method and location of fixation. 

Bone tunnels [44], keyholes [49], suture to a bed of decorticated bicipital groove, interference screws [44, 49–53], and suture anchors [49, 54, 55] are some of the several methods of fixation proximal or distal to the bicipital groove using open or arthroscopic techniques. Biomechanical studies have proved that the interference screws have the highest ultimate load to failure and the least amount of displacement on cyclic loading [44, 49, 54, 56].

Proximal fixation usually is performed under all-arthroscopic technique just proximal within the bicipital groove [46, 47, 51, 52, 58]. Some authors support that this location of fixation increases the rates of potential residual postoperative pain due to tenosynovitis within the biceps sheath [44, 59], leading to revision surgery. Advocates of distal fixation support that biceps muscle strength and length can be maintained without irritating the bicep's sheath inside the groove by fixing the tendon just outside the groove, either with arthroscopic or open techniques. Several open techniques have been also described, with the subpectoral miniopen being the most popular [19] and with few complications. The incidence of Popeye deformity is 0.2% and of persistent pain 0.2% approximately.

The shoulder rehabilitation protocol of a rotator cuff repair procedure is slightly altered after concomitant LHBT tenodesis. Usually progressive glenohumeral active and passive range of motion exercises are followed during the first 6 weeks. Active elbow flexion and supination exercises are also restricted during this postoperative period. Typically, patients with rotator cuff repair and biceps tenodesis are able to return to unrestricted activities after a period of four months [19].

Finally, numerous complications have been reported after proximal biceps tenodesis. Nho et al. [60] reported 0.7% complications rates. Patients had persistent bicipital pain or failed fixation with associated Popeye deformity (0.2%). One patient (0.1%) presented with wound infection, temporary musculocutaneous neuropathy, complex regional pain syndrome, and proximal humerus fracture. Individual cases of failure fixation [61] or humeral shaft fractures at the site of fixation have also been described recently [62].

### 6.3. Tenotomy versus Tenodesis

As mentioned previously, debate continues regarding the use of simple tenotomy instead of tenodesis for the treatment of LHBT lesions. Different studies have reported widely variable differences in the clinical outcomes of these two surgical methods of treatment, and no clearcut advantage of any one of those two treatment modalities has been proven to date [43–46]. However, no reports or analyses have demonstrated to date the reasons why simple tenotomy may result in such a broad spectrum of clinical outcomes and complications.

Additionally, there is no standard management algorithm of LHBT lesions due to lack of studies with high levels of evidence. Unofficially, a general approach suggests biceps tenotomy in patients over 55 to 60 years old, while heavy laborers and younger active patients may benefit from tenodesis [42]. Advantages and disadvantages of both techniques are presented in Table 1.

Numerous studies in the literature made an effort to compare these procedures, but controversy persists regarding the optimal course of surgical management (Table 2).

In general, tenodesis results in a good or excellent result in 74% of the cases, in cosmetic deformity (Popeye sign) in 8%, and in persistent pain in 24%. Tenotomy procedures result in good or excellent results in 77% of cases with 19% postoperative bicipital pain and 43% occurrence of Popeye sign [63].

However, the postoperative position and condition of the LHBT following simple tenotomy have not been adequately studied to date, nor have specific factors affecting the achievement or not of “natural tenodesis” been detected.

## 7. Summary

It must be our daily practice to inform all the patients about the two surgical options and the possible prevalence of cosmetic deformities, muscle cramps, or fatigue after tenotomy or pain at the groove after tenodesis. Additionally, patients undergoing tenodesis must be informed about the strict rehabilitation program, involving restriction of elbow flexion/extension and supination/pronation for 6 weeks after surgery [53]. When considering tenotomy versus tenodesis for the treatment of biceps lesions, literature guidelines [15, 41] should be followed but higher-level studies are needed in order to adopt a worldwide accepted algorithm treatment. 

## Figures and Tables

**Table 1:** Tenotomy versus tenodesis: advantages and disadvantages.

Tenotomy	Tenodesis
Advantages	

Simple procedure	Length-tension relation maintenance
Well-tolerated	Normal elbow flexion
Less rehabilitation protocol	Normal supination power
Faster return to activity	Minimize cosmetic deformity Avoid cramping pain

Disadvantages	

Cosmetic deformity (Popeye sign)	Longer rehabilitation
Cramping	More demanding procedure
Fatigue pain	Low rates of failure fixation, humeral
Loss of supination strength	Shaft fractures, CRPS, infection

**Table 2:** Summary of four articles on tenotomy versus tenodesis.

Study	Tenotomy (patients)	Tenodesis (patients)	Associated shoulder pathology	Popeye sign	Results
Osbahr et al. [45]	80	80	RCT AC Joint	No	Pain Tenotomy 65% Tenodesis 60%
Boileau et al. [23]	39	33	RCT	Yes	Satisfaction Tenotomy 72% Tenodesis 85%
Edwards et al. [57]	13	48	Subscapularis tears	N/A	Satisfaction Tenotomy 82% Tenodesis 80%
Koh et al. [42]	41	43	RCT	9% Tenodesis 27% Tenotomy	Satisfaction Tenotomy 85% Tenodesis 84%

## References

[1] Synder SJ, Karzel RP, Del Pizzo W, Ferkel RD, Friedman MJ (1990). SLAP lesions of the shoulder. *Arthroscopy*.

[2] Ahrens PM, Boileau P (2007). The long head of biceps and associated tendinopathy. *Journal of Bone and Joint Surgery B*.

[3] Gleason PD, Beall DP, Sanders TG (2006). The transverse humeral ligament: a separate anatomical structure or a continuation of the osseous attachment of the rotator cuff?. *American Journal of Sports Medicine*.

[4] Arai R, Mochizuki T, Yamaguchi K (2010). Functional anatomy of the superior glenohumeral and coracohumeral ligaments and the subscapularis tendon in view of stabilization of the long head of the biceps tendon. *Journal of Shoulder and Elbow Surgery*.

[5] Kask K, Põldoja E, Lont T (2010). Anatomy of the superior glenohumeral ligament. *Journal of Shoulder and Elbow Surgery*.

[6] Nakata W, Katou S, Fujita A, Nakata M, Lefor AT, Sugimoto H (2011). Biceps pulley: normal anatomy and associated lesions at MR arthrography. *Radiographics*.

[7] Werner A, Mueller T, Boehm D, Gohlke FE (2000). The stabilizing sling for the long head of the biceps tendon in the rotator cuff interval: a histoanatomic study. *American Journal of Sports Medicine*.

[8] Cheng NM, Pan WR, Vally F, Le Roux CM, Richardson MD (2010). The arterial supply of the long head of biceps tendon: anatomical study with implications for tendon rupture. *Clinical Anatomy*.

[9] Alpantaki K, McLaughlin D, Karagogeos D, Hadjipavlou A, Kontakis G (2005). Sympathetic and sensory neural elements in the tendon of the long head of the biceps. *Journal of Bone and Joint Surgery A*.

[10] Dierickx C, Ceccarelli E, Conti M, Vanlommel J, Castagna A (2009). Variations of the intra-articular portion of the long head of the biceps tendon: a classification of embryologically explained variations. *Journal of Shoulder and Elbow Surgery*.

[11] Kanatli U, Ozturk BY, Esen E, Bolukbasi S (2011). Intra-articular variations of the long head of the biceps tendon. *Knee Surgery, Sports Traumatology, Arthroscopy*.

[12] Vangsness CT, Jorgenson SS, Watson T, Johnson DL (1994). The origin of the long head of the biceps from the scapula and glenoid labrum. An anatomical study of 100 shoulders. *Journal of Bone and Joint Surgery B*.

[13] Patte D, Walch G, Boileau P (1990). Luxation de la longue portion du biceps et rapture de la cauffe des rotateurs. *Revue de Chirurgie Orthopedique*.

[14] Murthi AM, Vosburgh CL, Neviaser TJ (2000). The incidence of pathologic changes of the long head of the biceps tendon. *Journal of Shoulder and Elbow Surgery*.

[15] Walch G, Edwards TB, Boulahia A, Nové-Josserand L, Neyton L, Szabo I (2005). Arthroscopic tenotomy of the long head of the biceps in the treatment of rotator cuff tears: clinical and radiographic results of 307 cases. *Journal of Shoulder and Elbow Surgery*.

[16] Neviaser TJ, Neviaser RJ, Neviaser JS, Neviaser JS (1982). The four-in-one arthroplasty for the painful arc syndrome. *Clinical Orthopaedics and Related Research*.

[17] Miller C, Savoie FH (1994). Glenohumeral abnormalities associated with full-thickness tears of the rotator cuff. *Orthopaedic Review*.

[18] Refior HJ, Sowa D (1995). Long tendon of the biceps brachii: sites of predilection for degenerative lesions. *Journal of Shoulder and Elbow Surgery*.

[19] Nho SJ, Strauss EJ, Lenart BA (2010). Long head of the biceps tendinopathy: diagnosis and management. *Journal of the American Academy of Orthopaedic Surgeons*.

[20] Ahmad CS, DiSipio C, Lester J, Gardner TR, Levine WN, Bigliani LU (2007). Factors affecting dropped biceps deformity after tenotomy of the long head of the biceps tendon. *Arthroscopy*.

[21] Boileau P, Ahrens PM, Hatzidakis AM (2004). Entrapment of the long head of the biceps tendon: the hourglass biceps—a cause of pain and locking of the shoulder. *Journal of Shoulder and Elbow Surgery*.

[22] Post M, Benca P (1989). Primary tendinitis of the long head of the biceps. *Clinical Orthopaedics and Related Research*.

[23] Boileau P, Baqué F, Valerio L, Ahrens P, Chuinard C, Trojani C (2007). Isolated arthroscopic biceps tenotomy or tenodesis improves symptoms in patients with massive irreparable rotator cuff tears. *Journal of Bone and Joint Surgery A*.

[24] Walch G, Nové-Josserand L, Boileau P, Levigne C (1998). Subluxations and dislocations of the tendon of the long head of the biceps. *Journal of Shoulder and Elbow Surgery*.

[25] Chen CH, Hsu KY, Chen WJ, Shih CH (2005). Incidence and severity of biceps long head tendon lesion in patients with complete rotator cuff tears. *Journal of Trauma*.

[26] Chen C-H, Chen C-H, Chang C-H (2012). Classification and analysis of pathology of the long head of the biceps tendon in complete rotator cuff tears. *Chang Gung Medical Journal*.

[27] Holtby R, Razmjou H (2004). Accuracy of the Speed's and Yergason's tests in detecting biceps pathology and SLAP lesions: comparison with arthroscopic findings. *Arthroscopy*.

[28] Calvert E, Chambers GK, Regan W, Hawkins RH, Leith JM (2009). Special physical examination tests for superior labrum anterior posterior shoulder tears are clinically limited and invalid: a diagnostic systematic review. *Journal of Clinical Epidemiology*.

[29] Karlsson J (2010). Physical examination tests are not valid for diagnosing slap tears: a review. *Clinical Journal of Sport Medicine*.

[30] Tallia AF, Cardone DA (2003). Diagnostic and therapeutic injection of the shoulder region. *American Family Physician*.

[31] Hashiuchi T, Sakurai G, Morimoto M, Komei T, Takakura Y, Tanaka Y (2011). Accuracy of the biceps tendon sheath injection: ultrasound-guided or unguided injection? A randomized controlled trial. *Journal of Shoulder and Elbow Surgery*.

[32] Iannotti JP, Ciccone J, Buss DD (2005). Accuracy of office-based ultrasonography of the shoulder for the diagnosis of rotator cuff tears. *Journal of Bone and Joint Surgery A*.

[33] Papatheodorou A, Ellinas P, Takis F, Tsanis A, Maris I, Batakis N (2006). US of the shoulder: rotator cuff and non-rotator cuff disorders. *Radiographics*.

[34] Mohtadi NG, Vellet AD, Clark ML (2004). A prospective, double-blind comparison of magnetic resonance imaging and arthroscopy in the evaluation of patients presenting with shoulder pain. *Journal of Shoulder and Elbow Surgery*.

[35] Chang D, Mohana-Borges A, Borso M, Chung CB (2008). SLAP lesions: anatomy, clinical presentation, MR imaging diagnosis and characterization. *European Journal of Radiology*.

[36] Morag Y, Jacobson JA, Shields G (2005). MR arthrography of rotator interval, long head of the biceps brachii, and biceps pulley of the shoulder. *Radiology*.

[37] Barber FA, Byrd JWT, Wolf EM, Burkhart SS (2001). How would you treat the partially torn biceps tendon?. *Arthroscopy*.

[38] Sethi N, Wright R, Yamaguchi K (1999). Disorders of the long head of the biceps tendon. *Journal of Shoulder and Elbow Surgery*.

[39] Busconi BB, Deangelis N, Guerrero PE (2008). The proximal biceps tendon: tricks and pearls. *Sports Medicine and Arthroscopy Review*.

[40] Szabó I, Boileau P, Walch G (2008). The proximal biceps as a pain generator and results of tenotomy. *Sports Medicine and Arthroscopy Review*.

[41] Frost A, Zafar MS, Maffulli N (2009). Tenotomy versus tenodesis in the management of pathologic lesions of the tendon of the long head of the biceps brachii. *American Journal of Sports Medicine*.

[42] Koh KH, Ahn JH, Kim SM, Yoo JC (2010). Treatment of biceps tendon lesions in the setting of rotator cuff tears: prospective cohort study of tenotomy versus tenodesis. *American Journal of Sports Medicine*.

[43] Barber FA, Field LD, Ryu RK (2008). Biceps tendon and superior labrum injuries: decision making. *Instructional Course Lectures*.

[44] Mazzocca AD, Bicos J, Santangelo S, Romeo AA, Arciero RA (2005). The biomechanical evaluation of four fixation techniques for proximal biceps tenodesis. *Arthroscopy*.

[45] Osbahr DC, Diamond AB, Speer KP (2002). The cosmetic appearance of the biceps muscle after long-head tenotomy versus tenodesis. *Arthroscopy*.

[46] Romeo AA, Mazzocca AD, Tauro JC (2004). Arthroscopic biceps tenodesis. *Arthroscopy*.

[47] Kelly AM, Drakos MC, Fealy S, Taylor SA, O’Brien SJ (2005). Arthroscopic release of the long head of the biceps tendon: functional outcome and clinical results. *American Journal of Sports Medicine*.

[48] Gill TJ, McIrvin E, Mair SD, Hawkins RJ (2001). Results of biceps tenotomy for treatment of pathology of the long head of the biceps brachii. *Journal of Shoulder and Elbow Surgery*.

[49] Ozalay M, Akpinar S, Karaeminogullari O (2005). Mechanical strength of four different biceps tenodesis techniques. *Arthroscopy*.

[50] Gartsman GM, Hammerman SM (2000). Arthroscopic biceps tenodesis: operative technique. *Arthroscopy*.

[51] Kim SH, Yoo JC (2005). Arthroscopic biceps tenodesis using interference screw: end-tunnel technique. *Arthroscopy*.

[52] Lo IKY, Burkhart SS (2004). Arthroscopic biceps tenodesis using a bioabsorbable interference screw. *Arthroscopy*.

[53] Boileau P, Krishnan SG, Coste JS, Walch G (2002). Arthroscopic biceps tenodesis: a new technique using bioabsorbable interference screw fixation. *Arthroscopy*.

[54] Kilicoglu O, Koyuncu O, Demirhan M (2005). Time-dependent changes in failure loads of 3 biceps tenodesis techniques: In vivo study in a sheep model. *American Journal of Sports Medicine*.

[55] Richards DP, Burkhart SS (2005). A biomechanical analysis of two biceps tenodesis fixation techniques. *Arthroscopy*.

[56] Golish SR, Caldwell PE, Miller MD (2008). Interference screw versus suture anchor fixation for subpectoral tenodesis of the proximal biceps tendon: a cadaveric study. *Arthroscopy*.

[57] Edwards TB, Walch G, Sirveaux F (2006). Repair of tears of the subscapularis. Surgical technique. *The Journal of Bone and Joint Surgery*.

[58] Klepps S, Hazrati Y, Flatow E (2002). Arthroscopic biceps tenodesis. *Arthroscopy*.

[59] Friedman DJ, Dunn JC, Higgins LD, Warner JJP (2008). Proximal biceps tendon: injuries and management. *Sports Medicine and Arthroscopy Review*.

[60] Nho SJ, Reiff SN, Verma NN, Slabaugh MA, Mazzocca AD, Romeo AA (2010). Complications associated with subpectoral biceps tenodesis: low rates of incidence following surgery. *Journal of Shoulder and Elbow Surgery*.

[61] Koch BS, Burks RT (2012). Failure of biceps tenodesis with interference screw fixation. *Arthroscopy*.

[62] Sears BW, Spencer EE, Getz CL (2011). Humeral fracture following subpectoral biceps tenodesis in 2 active, healthy patients. *Journal of Shoulder and Elbow Surgery*.

[63] Slenker NR, Lawson K, Ciccotti MG, Dodson CC, Cohen SB (2012). Biceps tenotomy versus tenodesis: clinical outcomes. *Arthroscopy*.

